# Anti-Obesity Effect of α-Cubebenol Isolated from *Schisandra chinensis* in 3T3-L1 Adipocytes

**DOI:** 10.3390/biom11111650

**Published:** 2021-11-08

**Authors:** Su Jin Lee, Ji Eun Kim, Yun Ju Choi, Jeong Eun Gong, You Jeong Jin, Da Woon Lee, Young Whan Choi, Dae Youn Hwang

**Affiliations:** 1 Department of Biomaterials Science (BK21 FOUR Program), Life and Industry Convergence Research Institute, College of Natural Resources and Life Science, Pusan National University, Miryang 50463, Korea; nuit4510@naver.com (S.J.L.); prettyjiunx@naver.com (J.E.K.); poiu335@naver.com (Y.J.C.); jegog@naver.com (J.E.G.); hjinyuu1@naver.com (Y.J.J.); dawoon2989@kribb.re.kr (D.W.L.); 2 Department of Horticultural Bioscience, Life and Industry Convergence Research Institute, College of Natural Resources & Life Science, Pusan National University, Miryang 50463, Korea; ywchoi@pusan.ac.kr; 3 Longevity & Wellbeing Research Center, Laboratory Animals Resources Center, College of Natural Resources and Life Science, Pusan National University, Miryang 50463, Korea

**Keywords:** α-cubebenol, obesity, lipogenesis, β-oxidation, lipolysis, inflammasome, cytokines

## Abstract

The efficacy of α-cubebenol isolated from *Schisandra chinensis* has been studied in several diseases, including cecal ligation, puncture challenge-induced sepsis, and degranulation of neutrophils. To identify the novel functions of α-cubebenol on lipid metabolism, alterations on the regulation of lipogenesis, lipolysis, and inflammatory response were observed in 3T3-L1 adipocytes treated with α-cubebenol. Most lipogenic targets, including lipid accumulation, level of lipogenic transcription factors, and expression of lipogenic regulators, were suppressed in MDI (3-isobutyl-1-methylxanthine, dexamethasone, and insulin)-stimulated 3T3-L1 adipocytes treated with α-cubebenol without significant cytotoxicity. In addition, similar inhibition effects were observed in the iNOS-induced COX-2 mediated pathway and NLRP3 inflammasome pathway of MDI-stimulated 3T3-L1 cells treated with α-cubebenol. Lipolytic targets, such as cAMP concentration, expression of adenylyl cyclase and PDE4, and their downstream signaling pathway, in MDI-stimulated 3T3-L1 cells were stimulated by the α-cubebenol treatment. The levels of transcription factors and related proteins for β-oxidation were significantly higher in the MDI + α-cubebenol treated group than in the MDI + Vehicle treated group. These results show that α-cubebenol has a novel role as a lipogenesis inhibitor, lipolysis and β-oxidation stimulator, and inflammasome suppressor in MDI-stimulated 3T3-L1 adipocytes.

## 1. Introduction

The suitable balance between lipogenesis, lipolysis, and oxidation in lipid metabolism is well known as an important factor to maintain systemic energy homeostasis and insulin sensitivity [1]. Loss of this balance causes various metabolic disorders including obesity, diabetes, non-alcoholic fatty liver disease (NAFLD), and cardiovascular disease (CVD) [2]. Based on above scientific evidences, several mechanisms, including inhibition of lipogenesis, stimulation of lipolysis, and suppression of the inflammation response have been considered therapeutic targets for obesity [3,4,5,6,7]. In most studies evaluating new candidates for anti-obesity therapy, each target was controlled by a single compound derived from natural products. Some lipogenic targets, such as PPARγ, C/EBPα, Fas, and aP2, were suppressed in 3T3-L1 adipocytes after treatment with several compounds, including 6,6-Bieckol, diallyl sulfide, and platycodin D [8,9,10]. Aculeatin, biflavones, and resveratrol stimulate lipolysis targets by activating lipases and increasing glycerol release [11,12,13]. Furthermore, allin and saccharin inhibit the expression of inflammatory cytokines target, including IL-6 and IL-1β, during the anti-inflammatory response in 3T3-L1 adipocytes [14,15].

Few studies have focused on identifying a single bioactive compound that has beneficial effects on multiple targets for obesity treatment, even though this is a very economical and effective treatment strategy. Thus far, fucoidan, carsonic acid, capsaicin, and 6-shagol are well-known multi-targeting compounds derived from natural products [16,17,18,19,20]. Fucoidan, which is extracted from the sporophyll of *Undaria* *pinnatifida*, suppressed lipogenic targets, such as PPARγ, C/EBPα, Fas, and aP2, as well as downregulated the expression of inflammatory cytokines such as TNF-α, MCP-1, and PAI-1 [16]. In addition, carsonic acid, extracted from *Rosmarinus officinalis*, suppressed the adipogenic and inflammatory targets by regulating the NF-κB pathway [17]. Moreover, capsaicin, extracted from chili pepper, suppressed lipogenesis and lipolysis by upregulating lipases [18,19]. Finally, 6-shagaol suppressed lipogenesis by downregulating PPARγ and C/EBPα accelerating lipolysis and β-oxidation [20]. Therefore, it is essential to verify the molecular mechanism and therapeutic effects of novel bioactive compounds isolated from natural products that can control multiple targets for obesity.

α-Cubebenol is one of the cubebene series isolated from the extract of the dried fruits of *S. chinensis*. Although the compound is structurally very similar to cubebenoate, these two active compounds are known to have different therapeutic effects after the isolation by different extraction methods [21,22]. α-Cubebenol has therapeutic effects on various pathological conditions, including inflammation, neuroinflammation, cancer, and sepsis. α-Cubebenol effectively inhibits the production of nitric oxide (NO), expression of iNOS and COX-2 mRNA, nuclear translocation of NF-κB, and activation of MAPK signal in macrophages [23,24,25]. Similar inhibitory effects on neuroinflammation were observed in an Amyloid β1-42-treated BV-2 microglial cells and scopolamine (SC)-induced memory impairment model after an α-cubebenol treatment [26,27]. This compound induced p53-independent pathway-mediated apoptosis through the alternative regulation of Cas3, Bax, Bcl-2, and p53 proteins in hepatocellular carcinoma cells [28]. Furthermore, α-cubebenol inhibited immune cell apoptosis and the production of proinflammatory cytokines in a cecal ligation and puncture (CLP) challenge-induced sepsis model [29]. On the other hand, no studies have provided scientific evidence on the anti-obesity effects of α-cubebenol in pre- and mature adipocytes until now. Furthermore, we selected α-cubebenol as compound with potential anti-obesity effects because cubebenoate, which has a similar structure, showed excellent efficacy on the inhibition of lipogenesis and stimulation of lipolysis in adipocytes cells.

This study examined the novel role of α-cubebenol in regulating multiple targets on the lipid metabolism, including lipogenesis, lipolysis, and inflammatory responses in 3T3-L1 adipocytes. The results provide the first scientific evidence that the anti-obesity effects of α-cubebenol may be closely associated with the inhibition of lipogenesis, stimulation of lipolysis, and suppression of the inflammatory response in 3T3-L1 adipocytes.

## 2. Materials and Methods

### 2.1. Purification of α-Cubebenol

**Figure 1:**
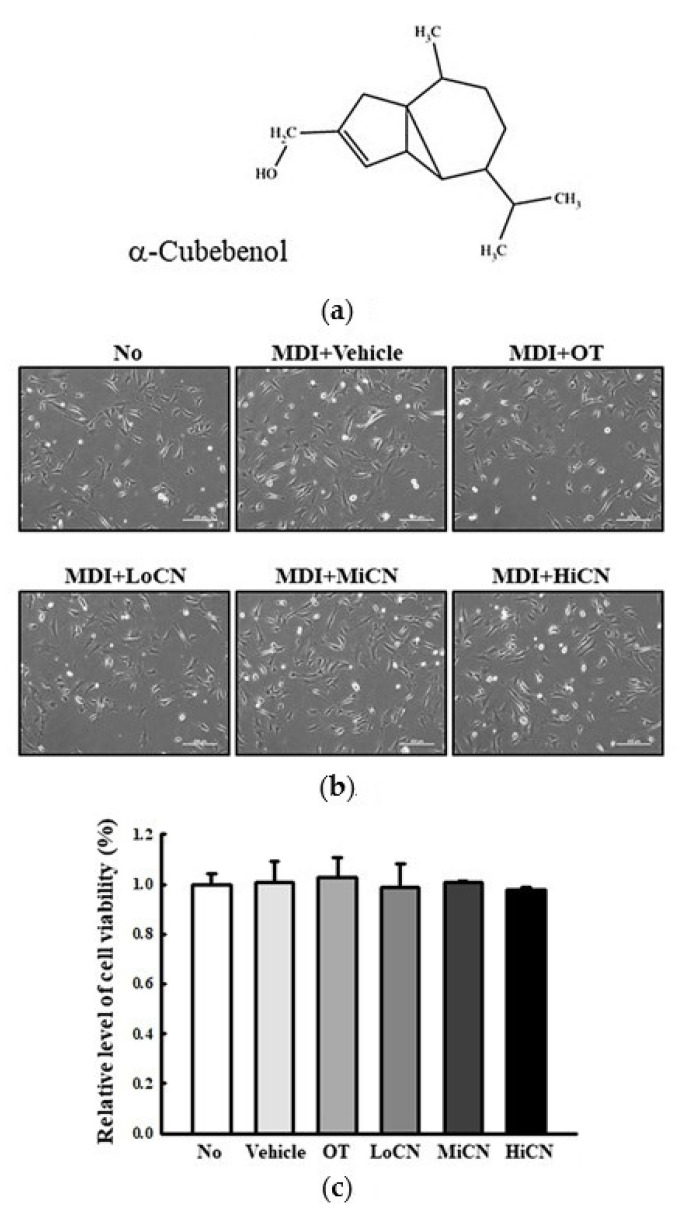
Cytotoxicity. (**a**) Chemical structure of α-cubebenol. (**b**,**c**) Viability of 3T3-L1 pre-adipocytes against α-cubebenol. After the treatment of 7.5, 15, and 30 μg/mL of α-cubebenol for 24 h, cell morphological changes were observed under a microscope at 200 × magnification, and the cell viability was determined using the MTT assay. Three wells per group were used for the MTT assay, and optical density was measured in triplicates. The data represents the means ± SD of triplicates. OT, orlistat; MDI, adipogenic cocktail consisting of 3-isobutyl-1-methylxanthine, dexamethasone, and insulin; LoCN, low concentration (7.5 μg/mL) of α-cubebenol; MiCN, middle concentration (15 μg/mL) of α-cubebenol; HiCN, high concentration (30 μg/mL) of α-cubebenol.

α-Cubebenol (Figure 1a) was isolated and purified as described elsewhere [23]. Briefly, the fruit of *S. chinensis* (Turcz.) Baill was collected in September 2010 in Moonkyong, South Korea, and identified by Professor Young Whan Choi, Department of Horticultural Bioscience, Pusan National University. A voucher specimen (accession no. SC-PDRL-2) was deposited in the Herbarium at the Pusan National University. The dried fruits of *S. chinensis* (2.5 kg) were ground to a fine powder and extracted successively at room temperature with *n*-hexane, CHCl_3_, and MeOH. The hexane extract (308 g) was evaporated in vacuo and chromatographed on a silica gel column (100 × 10 cm) (40 μm; Baker, Phillipsburg, NJ, USA) with a step gradient (0%, 5%, and 20%) of EtOAc in hexane and 5% MeOH in CHCl_3_ to obtain 38 fractions, as described previously [23]. Fraction 9 (KH_9_, 4866 mg) was separated on a silica gel column (100 × 3.0 cm) with a step gradient (1%, 10%, and 15%) of acetone in CHCl_3_, to obtain 21 fractions. Fraction 2 (KH_9_IG, 529.9 mg) was separated on a silica gel column (100 × 3 cm) with 5% acetone in CHCl_3_ to yield α-cubebenol (162.9 mg), as described previously [23]. Pure α-cubebenol was identified by HPLC on a Luna C18 column (150 × 4.6 mm internal diameter (ID); 5-μM particle size; Phenomenex, Los Angeles, CA, USA) with a methanol–acetonitrile gradient at a flow rate of 1.0 mL/min. The structure of α-cubebenol isolated from *S. chinensis* fruits was identified by the _1_H and _13_C nuclear magnetic resonance (NMR) spectra (including Dept, heteronuclear single-quantum correlation, and heteronuclear multiple-bond correlation spectroscopy) in CDCl_3_, as described previously [23]. Finally, α-cubebenol was dissolved in a dimethyl sulfoxide (DMSO) solution (Duchefa Biochemie, Haarlem, The Netherlands) for further treatment.

### 2.2. Cell Culture, Adipocyte Differentiation, and Treatment of α-Cubebenol

The differentiation of adipocytes and treatment of α-cubebenol were performed as described in previous study [30]. Murine adipocyte 3T3-L1, used in this study, were obtained from the American Type Culture Collection (ATCC, Mannassas, VA, USA). They were cultured in Dulbecco Modified Eagle’s Medium (DMEM, Welgene, Gyeongsan-si, Korea) supplemented with 10% fetal bovine serum (FBS, Welgene), L-glutamine, penicillin, and streptomycin (Thermo Scientific, Waltham, MA, USA), as well as incubated in a humidified incubator at 37 °C under 5% CO_2_ and 95% fresh air.

Differentiation from pre-adipocytes to adipocytes was induced by the previously proposed method [30]. When 3T3-L1 pre-adipocyte were confluent up to 80–90% (differentiation day 0), normal culture media was removed from culture plate, and differentiation media (MDI) including 3-isobutyl-1-methylxanthine (0.5 mM, Sigma-Aldrich Co., St. Louis, MO, USA), dexamethasone (1 μM, Sigma-Aldrich Co.), and insulin (5 μg/mL, Sigma-Aldrich Co.) in DMEM supplemented with 10% fetal bovine serum (FBS) was added. After incubation for 2 days (differentiation day 2), MDI media was replaced by DMEM supplemented with 10% FBS and 5 μg/mL of insulin for two more days (differentiation day 4). Finally, the cells were maintained in DMEM supplemented with 10% FBS for four more days (differentiation day 8) (Supplementary Figure S1).

The suppressive effects of α-cubebenol on lipogenesis were examined as described in previous studies [30]. The MDI-treated 3T3-L1 cells (day 0) were classified into five groups, treated with the Vehicle (DMSO), orlistat (OT, 40 μg/mL, Sigma-Aldrich Co.), 7.5 μg/mL of α-cubebenol (LoCN, Low concentration of α-cubebenol), 15 μg/mL of α-cubebenol (MiCN, Medium concentration of α-cubebenol), or 30 μg/mL of α-cubebenol (HiCN, High concentration of α-cubebenol) during eight days of differentiation (from day 0 to 8) (Supplementary Figure S1). To examine lipolysis, differentiated 3T3-L1 adipocytes were treated with the same concentration of α-cubebenol for two days after differentiation (from day 8 to day 10) (Supplementary Figure S1). Finally, the cells of each group were harvested and used for further analyses.

### 2.3. Cell Viability Assay

The viability of 3T3-L1 pre-adipocytes was measured by the method using the 3-[4,5-dimethylthiazol-2-yl]-2,5 diphenyltetrazolium bromide (MTT) assay (Sigma-Aldrich Co.) as described in previous study [30]. α-Cubebenol concentrations (30 μg/mL = 136 μM) for this assay were determined based on the results from previous studies on the anti-tumor effects against hepatocellular carcinoma cells [28]. In the previous study, any significant toxicity was not detected at concentration below 160 uM of α-cubebenol [28]. Briefly, 1 × 10^4^ cells/0.2 mL of 3T3-L1 pre-adipocytes were seeded into 96 well plates and incubated for 24 h. When 3T3-L1 pre-adipocytes were confluent up to 70–80% in 96-well plates, they were treated with the vehicle (DMSO), OT (40 μg/mL, Sigma-Aldrich Co.), 7.5 μg/mL α-cubebenol (LoCN, Low concentration of α-cubebenol), 15 μg/mL α-cubebenol (MiCN, Medium concentration of α-cubebenol), or 30 μg/mL α-cubebenol (HiCN, High concentration of α-cubebenol). After incubation for 24 h, 48 h, and 72 h in a 37 °C incubator, the supernatants of the 3T3-L1 pre-adipocytes in each well were replaced with MTT mixture consisting of fresh DMEM media (200 μL) and MTT solution (50 μL, 20 mg/mL in PBS). Then, the cells were incubated at 37 °C for 4 h, after which the precipitated formazan was dissolved in 150 μL of DMSO and the absorbance was read at 570 nm using a VERSA max Plate reader (Molecular Devices, Sunnyvale, CA, USA).

### 2.4. Oil Red O (ORO) Staining Analysis

The amount of accumulated lipid droplet in 3T3-L1 adipocytes was measured using ORO staining solution as described in a previous study [31]. Briefly, fully differentiated 3T3-L1 adipocytes of the subset group were fixed with 10% formalin for 10 min and washed three times with distilled water. Then, the cells were stained with 0.5% ORO dye solution (Sigma-Aldrich Co.) in 100% isopropanol (Sigma-Aldrich Co.) for 30 min at room temperature. Finally, the ORO-stained fat droplets were observed microscopically at 200× and 400× magnification (Leica Microsystems, Wetzlar, Germany) and the intensity of the color was measured using VERSA max Plate reader (Molecular Devices) after dissolving with 100% isopropanol.

### 2.5. Quantitative Reverse Transcription-Polymerase Chain Reaction (RT-qPCR) Analysis

The transcription levels of the lipid metabolism related genes were quantified with RT-qPCR as described in a previous study [32]. Briefly, total RNA of the subset groups was extracted with RNAzol (Tel-Test Inc., Friendswood, TX, USA) from 3T3-L1 adipocytes according to manufacturer’s guideline. Total RNA molecules were quantified using NanoDrop system (Shimadzu Biotech, Kyoto, Japan) and the synthesis of complement DNA using a mixture of total RNA (5 μg), oligo-dT primer (Invitrogen, Carlsbad, CA, USA), dNTP, and reverse transcriptase (Superscript II, Invitrogen) was conducted using T100 ™ thermal cycler (Bio-Rad, Hercules, CA, US). With the synthesized cDNA template, qPCR was conducted using 2× Power SYBR Green (Toyobo Co., Osaka, Japan) with the following cycles: 15 s at 95 °C, 30 s at 55 °C, and 60 s at 70 °C. The primer sequences for target gene expression identification are stated as table (Supplementary Table S1). The reaction cycle during which the PCR products exceeded this fluorescence intensity threshold during the exponential phase of PCR amplification was considered the threshold cycle (Ct). The expression of the target gene was quantified relative to that of the housekeeping gene β-actin, based on a comparison of the Ct values at a constant fluorescence intensity according to Livak and Schmittgen’s method [33].

### 2.6. Western Blot Analysis

The expression levels of the lipid metabolism related proteins were quantified with the Western blot assay as described in a previous study [30]. Total proteins were extracted from 3T3-L1 adipocytes using Pro-Prep Protein Extraction Solution (iNtRON Biotechnology, Seongnam, Korea) and quantified with SMARTTM BCA Protein Assay Kit (Thermo Scientific). An appropriate amount of protein (30 μg) were collected from total cell protein, and loaded equally and separated by 4–20% sodium dodecyl sulfate–polyacrylamide gel electrophoresis (SDS-PAGE) for 2 h, after which the resolved proteins were transferred to nitrocellulose membranes at 40 V for 2 h. Then, each membrane was incubated separately overnight at 4 °C with the following primary antibodies, diluted in 1:1000 (Supplement Table S2). The probed membranes were then washed with a washing buffer (137 mM NaCl, 2.7 mM KCl, 10 mM Na_2_HPO_4_, and 0.05% Tween 20) and incubated with 1:2000 diluted horseradish peroxidase (HRP)-conjugated goat anti-rabbit IgG (Invitrogen) at room temperature for 1 h. Finally, the membrane blots were developed using the Amersham ECL Select Western Blotting detection reagent (GE Healthcare, Little Chalfont, UK). The chemiluminescence signals that originated from the specific bands were detected using FluorChemi ^®^ FC2 (Alpha Innotech Co., San Leandro, CA, USA).

### 2.7. ELISA for cAMP Concentration

The cAMP concentration was determined using a cAMP ELISA kit (Cell Biolabs INC., San Diego, CA, USA) based on the manufacturer’s instructions. Briefly, the cell lysates were collected from 3T3-L1 adipocytes treated with the MDI + Vehicle, MDI + OT, and MDI + α-cubebenol, respectively. After centrifugation at 3000 rpm for 3 min, each sample was mixed with the labeled AP-conjugate and cAMP complete antibody. The pNpp substrate was added to each well and incubated for 1 h. Finally, the stop solution was added, and the absorbance of each well was read at 540 nm using a Vmax plate reader (Molecular Devices).

### 2.8. Measurement of Free Glycerol Release

The free glycerol release from the 3T3-L1 adipocytes was determined using a Cell-based Glycerol assay kit (Abcam), as described in a previous study [30]. Briefly, cell supernatants were collected from 3T3-L1 adipocytes treated with MDI + Vehicle, MDI + OT, and MDI + α-cubebenol, respectively. Subsequently, these samples (25 μL) were mixed with free glycerol reagent (100 μL) and incubated for 15 min at room temperature. Finally, the absorbance of the mixture was observed at 540 nm using a Vmax plate reader (Molecular Devices). The free glycerol concentration was calculated using the following equation:

Free glycerol (μg/mL) = A540-(y-intercept)/Slope(1)

### 2.9. Statistical Significance Analysis

The statistical significance was evaluated using a one-way analysis of variance (ANOVA) (SPSS for Windows, Release 10.10, Standard Version, Chicago, IL, USA) followed by Tukey’s post hoc t-test for multiple comparisons. All data are expressed as the means ± SD. A *p* value less than 0.05 was considered statistically significant.

## 3. Results

### 3.1. Determination of the Optimal Concentration of α-Cubebenol in 3T3-L1 Adipocytes

The optimal concentration of α-cubebenol to treat 3T3-L1 pre-adipocytes was determined. The cell viability of 3T3-L1 pre-adipocytes was measured after treatment with 10, 20, 30, 40, and 50 μg/mL of α-cubebenol. The cell viability of 3T3-L1 pre-adipocytes decreased significantly in only the 40 and 50 μg/mL α-cubebenol treated groups, while other groups maintained a constant level (Supplement Figure S1). Therefore, the optimal concentration of α-cubebenol was determined to be 7.5, 15, or 30 μg/mL based on the above cell viability test. Furthermore, at these concentrations for 24 h, 3T3-L1 pre-adipocytes showed a constant cell viability compared to the vehicle-treated group. The cell morphology completely reflected the cell viability (Figure 1). Moreover, these levels of cell viabilities were maintained for 72 h without any significant changes (Supplement Figure S2), while apoptotic cells were not detected at 24, 48, and 72 h after α-cubebenol treatment (Supplement Figure S3). Hence, the optimal concentration of α-cubebenol to evaluate its anti-obesity effect in 3T3-L1 pre-adipocytes is less than 30 μg/mL.

### 3.2. Inhibitory Effects of α-Cubebenol on Lipid Accumulation

**Figure 2:**
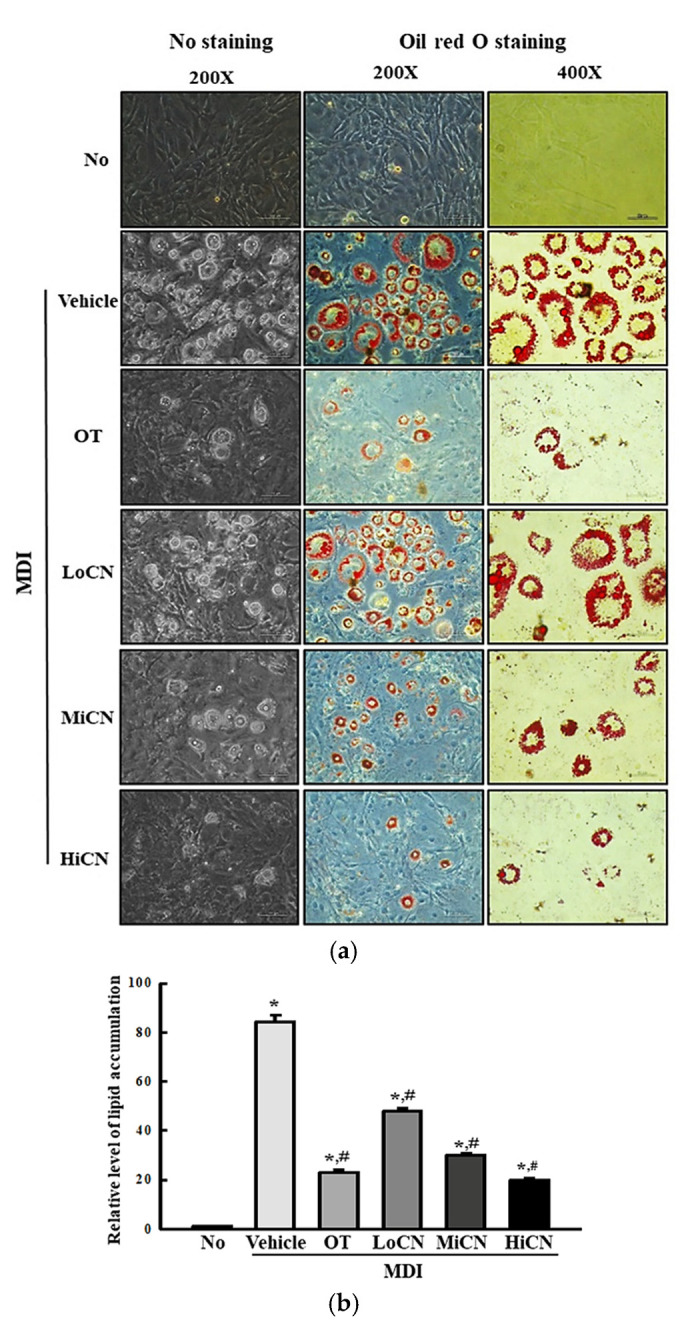
ORO staining analysis. (**a**) After inducing the differentiation with MDI media and treatment of α-cubebenol for eight days, 3T3-L1 adipocytes were stained with ORO dye solution. The images of the ORO-stained cells were observed under a microscope 200× and 400× magnification. (**b**) The optical density of red color was measured in the ORO-stained cells. Two to three wells per group were used for staining, and optical density was measured in duplicates. The data represent the means ± SD of duplicates. * indicates *p* < 0.05 compared to the No treated group. ^#^ indicates *p* < 0.05 compared to the MDI + Vehicle treated group. ORO, Oil Red O; OT, orlistat; MDI, adipogenic cocktail consisting of 3-isobutyl-1-methylxanthine, dexamethasone, and insulin; LoCN, low concentration (7.5 μg/mL) of α-cubebenol; MiCN, middle concentration (15 μg/mL) of α-cubebenol; HiCN, high concentration (30 μg/mL) of α-cubebenol.

To determine if α-cubebenol can suppress lipid accumulation during the differentiation of 3T3-L1 adipocytes, the amount of lipid droplets stained with ORO were measured in 3T3-L1 adipocytes cotreated with MDI and α-cubebenol for eight days. The relative level of lipid accumulation was elevated significantly in MDI + Vehicle treated group compared to the No treated group. On the other hand, these levels decreased remarkably after the α-cubebenol treatment in a dose-dependent manner. The highest level was detected in the MDI + HiCN treated group (Figure 2). These results demonstrate that α-cubebenol can inhibit the lipid accumulation induced by MDI in 3T3-L1 adipocytes.

### 3.3. Effects of α-Cubebenol on the Regulation of Lipogenic Genes Trascription

**Figure 3:**
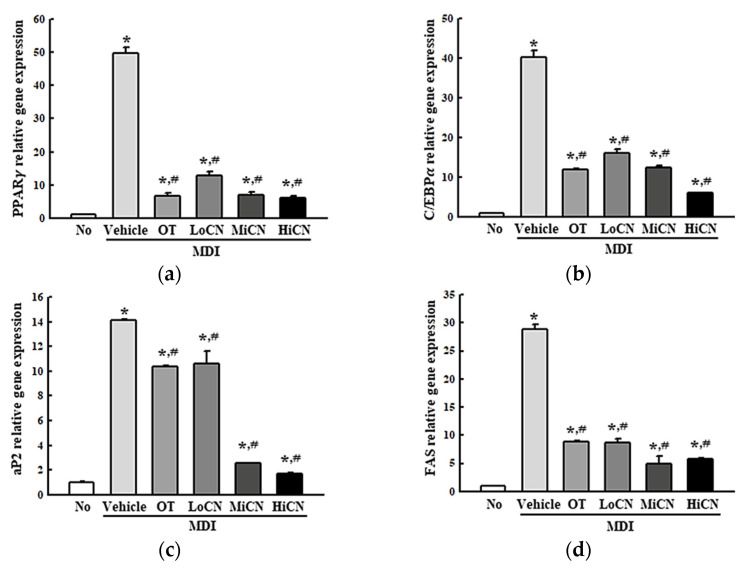
mRNA levels of adipogenic and lipogenic factors. After collecting the total RNA from MDI stimulated 3T3-L1 adipocytes treated with α-cubebenol, the mRNA levels of two adipogenic transcription factors (PPARγ (**a**) and C/EBPα (**b**) and two lipogenic regulators (aP2 (**c**) and FAS (**d**)) were quantified by RT-qPCR, as described in materials and methods. Two to three dishes per group were used to prepare the total RNAs, and qRT-PCR was assayed in duplicate for each sample. The data represent the means ± SD of duplicates. * indicates *p* < 0.05 compared to the No treated group. ^#^ indicates *p* < 0.05 compared to the MDI + Vehicle treated group. OT, orlistat; MDI, adipogenic cocktail consisting of 3-isobutyl-1-methylxanthine, dexame-thasone, and insulin; LoCN, low concentration (7.5 μg/mL) of α-cubebenol; MiCN, middle concentration (15 μg/mL) of α-cubebenol; HiCN, high concentration (30 μg/mL) of α-cubebenol.

The inhibitory effects of α-cubebenol on lipid accumulation accompanied by alternative regulation of lipogenic genes were examined. After the cotreatment of MDI media and α-cubebenol in the 3T3-L1 adipocytes for eight days, the transcription level of two transcription factors (PPARγ and C/EBPα) and two regulators (aP2, FAS), which participate in lipogenesis, were measured. During MDI-induced differentiation of 3T3-L1 adipocytes, the levels of PPARγ, C/EBPα, aP2, and FAS were remarkably increased compared to the No-treated group. By contrast, the elevated level in the differentiated 3T3-L1 adipocytes decreased significantly after the treatment of α-cubebenol (Figure 3). These levels in the MDI + OT treated group were similar to those of the MDI + LoCN treated group (Figure 3). Therefore, the inhibitory effect of α-cubebenol on lipid accumulation may be linked to the alterative regulation of lipogenic proteins.

### 3.4. Suppressive Effect of α-Cubebenol on the Inflammatory Response

**Figure 4:**
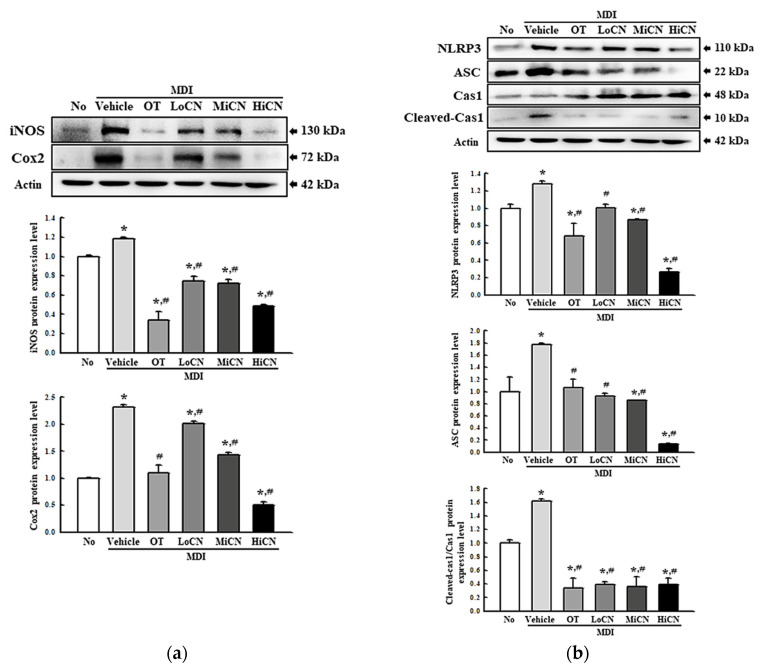
Expression level analyses for inflammatory markers. (**a**) Expression of COX-2 and iNOS proteins. After collecting the total proteins from MDI-stimulated 3T3-L1 adipocytes treated with α-cubebenol, the expression level of iNOS, COX-2, and β-actin were detected using the specific primary antibodies, followed by horseradish peroxidase (HRP)-conjugated goat anti-rabbit IgG. The intensity of each band was measured by an imaging densitometer, and the relative levels of each protein were calculated relative to the intensity of the actin bands. (**b**) Expression of inflammasome proteins. After collecting total proteins from MDI-stimulated 3T3-L1 adipocytes treated with α-cubebenol, the levels of NLRP3, ASC, Cas-1, Cleaved Cas-1, and β-actin expression were measured by Western blot analysis using the specific antibodies and HRP-conjugated anti-rabbit IgG antibody. Two to three dishes per group were used to prepare cell homogenates, and Western blot analysis was assayed in duplicate for each sample. The data represent the means ± SD of three replicates. * indicates *p* < 0.05 compared to the No treated group. ^#^ indicates *p* < 0.05 compared to the MDI + Vehicle treated group. OT, orlistat; MDI, adipogenic cocktail consisting of 3-isobutyl-1-methylxanthine, dexamethasone, and insulin; LoCN, low concentration (7.5 μg/mL) of α-cubebenol; MiCN, middle concentration (15 μg/mL) of α-cubebenol; HiCN, high concentration (30 μg/mL) of α-cubebenol.

**Figure 5:**
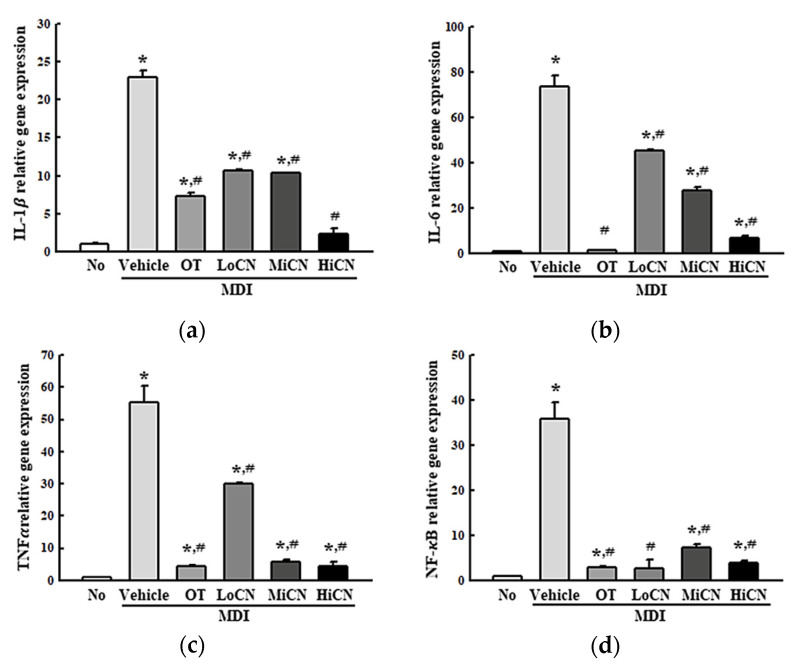
mRNA level of inflammatory cytokines. After collecting the total RNA from MDI stimulated 3T3-L1 adipocytes treated with α-cubebenol, the levels of IL-1β (**a**), IL-6 (**b**), TNF-α (**c**), and NF-κB (**d**) mRNA were quantified by RT-qPCR using the specific primers. Two to three dishes per group were used to prepare the total RNAs, and qRT-PCR was assayed in duplicate for each sample. The data represent the means ± SD of three replicates. * indicates *p* < 0.05 compared to the No treated group. ^#^ indicates *p* < 0.05 compared to the MDI + Vehicle treated group. OT, orlistat; MDI, adipogenic cocktail consisting of 3-isobutyl-1-methylxanthine, dexamethasone, and insulin; LoCN, low concentration (7.5 μg/mL) of α-cubebenol; MiCN, middle concentration (15 μg/mL) of α-cubebenol; HiCN, high concentration (30 μg/mL) of α-cubebenol.

This study examined whether the inhibitory effect of α-cubebenol on lipid accumulation was accompanied by an alteration in the inflammatory response. Hence, alterations of the iNOS-induced COX-2 mediated pathway and the expression levels of the NLR family pyrin domain containing 3 (NLRP3), apoptosis-associated speck-like protein containing a CARD (ASC), and Cleaved Cas-1 in the MDI-stimulated 3T3-L1 adipocytes after an α-cubebenol treatment were measured. The levels of iNOS and COX-2 expression were significantly higher in the MDI + Vehicle treated group than in the No treated group. On the other hand, these expression levels decreased remarkably in a dose-dependent manner after a treatment with α-cubebenol (Figure 4a). In addition, significant inhibition effects were observed in the NLRP3 inflammasome pathway. The expression levels of NLRP3, ASC proteins, and cleaved Cas-1 were significantly lower in the MDI + α-cubebenol-treated 3T3-L1 adipocytes than in the MDI + Vehicle treated group (Figure 4b). Furthermore, the above alterations were reflected in the expression of inflammatory cytokines, including TNF-α, IL-6, IL-1β, and NF-κ B. These levels of four cytokines were decreased in the MDI-induced differenced 3T3-L1 adipocytes after a treatment of α-cubebenol (Figure 5a–d). Therefore, the inhibitory effect of α-cubebenol on lipid accumulation may associate with suppression of the iNOS-induced COX-2 mediated pathway, NLRP3 inflammasome pathway, and expression of inflammatory cytokines.

### 3.5. Effects of α-Cubebenol on the Stimulation of Lipolysis

**Figure 6:**
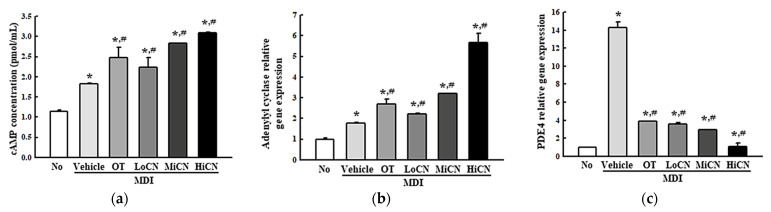
cAMP-mediated pathway analyses. (**a**) Relative level of cAMP concentration. After collecting the cell lysates from MDI-stimulated 3T3-L1 adipocytes treated with α-cubebenol, the cAMP concentration was measured using an ELISA assay. Two to three wells per group were used to collect the cell lysates, and the assay was measured in duplicate. (**b**,**c**) cAMP regulators analysis. After collecting the total RNA from MDI-stimulated 3T3-L1 adipocytes treated with α-cubebenol, the mRNA levels of adenylyl cyclase (**b**) and PDE4 (**c**) were quantified by RT-qPCR, as described in Materials and Methods. Two to three dishes per group were used to prepare the total RNAs, and qRT-PCR were assayed in duplicate for each sample. The data represent the means ± SD of three replicates. * indicates *p* < 0.05 compared to the No treated group. ^#^ indicates *p* < 0.05 compared to the MDI + Vehicle treated group. OT, orlistat; MDI, adipogenic cocktail consisting of 3-isobutyl-1-methylxanthine, dexamethasone, and insulin; LoCN, low concentration (7.5 μg/mL) of α-cubebenol; MiCN, middle concentration (15 μg/mL) of α-cubebenol; HiCN, high concentration (30 μg/mL) of α-cubebenol.

The alteration in cAMP concentration, the expression levels of the lipolytic proteins, and the free glycerol concentration were measured in differentiated 3T3-L1 adipocytes treated with α-cubebenol for two days to determine whether α-cubebenol is also associated with lipolytic effects. The cAMP concentration was enhanced significantly in the MDI + α-cubebenol-treated 3T3-L1 adipocytes compared to the MDI + Vehicle treated group (Figure 6a). In addition, a similar pattern was observed in the expression level of adenylyl cyclase (Figure 6b). On the other hand, a reversed regulation pattern was detected in PDE4 expression. The increased level of PDE4 expression was decreased remarkably in the MDI + α-cubebenol treated group, but the rate of the decrease varied (Figure 6c).

**Figure 7:**
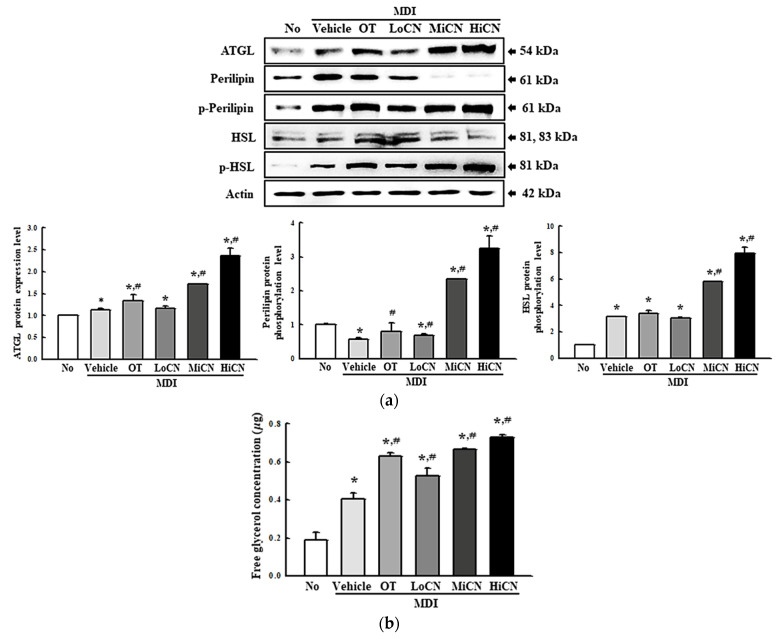
Expression of the lipolytic factors and the concentration of released free glycerol. (**a**) Expression level of the lipolytic proteins. After collecting the total proteins from the MDI-stimulated 3T3-L1 adipocytes treated with α-cubebenol, the expression level of ATGL, HSL, *p*-HSL, perilipin, *p*-perilipin, and β-actin were detected using the specific primary antibodies, followed by horseradish peroxidase (HRP)-conjugated goat anti-rabbit IgG. The intensity of each band was measured by an imaging densitometer, and the relative levels of each protein were calculated relative to the intensity of the actin bands. Two to three dishes per group were used to prepare the cell homogenates, and Western blot analysis was assayed in duplicate for each sample. (**b**) Level of free glycerol in culture media. The level of released glycerol was measured in the supernatant of the 3T3-L1 adipocytes treated with three different concentrations of α-cubebenol. Two to three wells per group were used for the assay, and the optical density was measured in duplicate. The data represent the means ± SD of three replicates. * indicates *p* < 0.05 compared to the No treated group. ^#^ indicates *p* < 0.05 compared to the MDI + Vehicle treated group. OT, orlistat; MDI, adipogenic cocktail consisting of 3-isobutyl-1-methylxanthine, dexamethasone, and insulin; LoCN, low concentration (7.5 μg/mL) of α-cubebenol; MiCN, middle concentration (15 μg/mL) of α-cubebenol; HiCN, high concentration (30 μg/mL) of α-cubebenol.

Moreover, the expression level of ATGL, perilipin, *p*-perilipin, HSL, and *p*-HSL were measured in differentiated 3T3-L1 adipocytes treated with α-cubebenol for two days to determine if the elevation of cAMP concentration was accompanied by an alteration on their downstream signaling pathway. The level of ATGL expression increased in a dose-dependent manner in the α-cubebenol treated MDI-stimulated 3T3-L1 adipocytes; besides, these levels were higher in the MiCN and HiCN-treated group than the in OT-treated group (Figure 7a). A similar increase pattern was detected in the phosphorylation of perilipin and HSL. These levels were increased remarkably in the MDI + MiCN and MDI + HiCN-treated groups compared to the MDI + Vehicle treated group. On the other hand, this increase was not observed in the MDI + LoCN-treated group (Figure 7a). Furthermore, the free glycerol concentration was measured in a cultured medium of MDI + α-cubebenol-treated 3T3-L1 adipocytes to confirm the production of the final products. The free glycerol concentration in the treated 3T3-L1 adipocyte cultured medium was increased significantly in a dose-dependent manner (Figure 7b). These results suggest that α-cubebenol can stimulate lipolysis by regulating the cAMP signaling pathway.

### 3.6. Stimulatory Effects of α-Cubebenol on the β-Oxidation of Lipid

**Figure 8:**
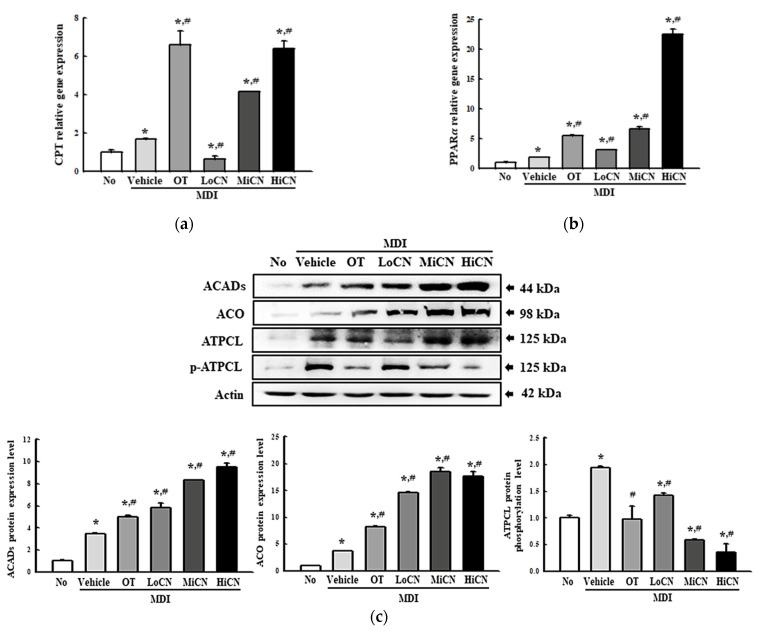
mRNA levels and protein expression of the 𝛽-oxidation relative factors. (**a**,**b**) mRNA levels of the 𝛽-oxidation relative factors. After collecting the total RNA from MDI stimulated 3T3-L1 adipocytes treated with α-cubebenol, the mRNA levels of CPT (**a**) and PPARα (**b**) gene for 𝛽-oxidation were measured by RT-qPCR, as described in Materials and Methods. Two to three dishes per group were used to prepare the total RNAs, and qRT-PCR were assayed in duplicate for each sample. (**c**) Expression level of 𝛽-oxidation relative proteins. After collecting the total proteins from the MDI-stimulated 3T3-L1 adipocytes treated with α-cubebenol, the expression level of ACADS, ACO, ATPCL, *p*-ATPCL, and β-actin were detected using the specific primary antibodies, followed by horseradish peroxidase (HRP)-conjugated goat anti-rabbit IgG. The intensity of each band was measured by an imaging densitometer, and the relative levels of each protein were calculated relative to the intensity of the actin bands. Two to three dishes per group were used to prepare the cell homogenates, and Western blot analysis was assayed in duplicate for each sample. The data represent the means ± SD of three replicates. * indicates *p* < 0.05 compared to the No treated group. ^#^ indicates *p* < 0.05 compared to the MDI + Vehicle treated group. OT, orlistat; MDI, adipogenic cocktail consisting of 3-isobutyl-1-methylxanthine, dexamethasone, and insulin; LoCN, low concentration (7.5 μg/mL) of α-cubebenol; MiCN, middle concentration (15 μg/mL) of α-cubebenol; HiCN, high concentration (30 μg/mL) of α-cubebenol.

Finally, this study investigated whether α-cubebenol can stimulate β-oxidation of lipid in MDI-stimulated 3T3-L1 adipocytes. The alterations in the level of a transcription factor (PPARα) and β-oxidation related proteins (CPT, ACADs, ACO, ATPCL, and *p*-ATPCL) were measured in differentiated 3T3-L1 adipocytes treated with α-cubebenol for two days. The mRNA expression of PPARα was increased remarkably in the MDI + LoCN, MDI + MiCN, and MDI + HiCN treated groups compared with the MDI + Vehicle-treated group (Figure 8a). A similar pattern was detected in CPT expression, but the OT-treated group showed a different pattern (Figure 8b). Furthermore, a significant increase in the expression level of two β-oxidation-related proteins (ACADs and ACO) was detected after the α-cubebenol treatment. On the other hand, the level of ATPCL phosphorylation decreased in a dose-dependent manner in MDI-stimulated 3T3-L1 adipocytes after the α-cubebenol treatment (Figure 8c). These results suggest that α-cubebenol can stimulate β-oxidation through the alternative regulation of the PPARα and β-oxidation related proteins.

## 4. Discussion

Most studies examining the role of α-cubebenol focused on the therapeutic effects on single targets of the physiological metabolism in several diseases, such as sepsis and cancer [26,27]. Hence, an examination of the role of a single compound with efficacy in multiple targets is very important for overcoming these limitations. As part of this research, this study investigated the therapeutic effects and molecular mechanism of α-cubebenol on lipogenesis, lipolysis, and inflammation in 3T3-L1 adipocytes. These results showed that α-cubebenol has potential as an anti-obesity drug, but further research will be needed in animal models.

Until now, various bioactive compounds such as lignans, triterpenes, flavonoids, essential oils, phenolic acids, and polysaccharides were isolated from the leaves, shoots, and seeds of *S. chinensis* [34,35,36]. Among these, compounds of cubebene series including cubebene, cubebenoate, and cubeneol have recently received great attention as a newly isolated active compound, although there are differences in the extraction method and chemical structure [21,22]. In particular, cubenenol used in this study is structurally similar to cubebenoate, although there are many differences in their functions [24,25,26,27,28,29]. The above two compounds show similar therapeutic effects in inflammation and sepsis, although each one has different therapeutic effects in various diseases [24,29,37,38]. Several beneficial effects of cubebenoate were reported in chronic diseases. Cubebenoate has anti-inflammatory effects in mouse peritoneal macrophages through the regulation of inducible nitric oxide synthase (iNOS) and cyclooxygenase (COX)-2, and the production of nitric oxide (NO) and prostaglandin E2 (PGE2) [39]. Moreover, this compound induced the blockade of lung inflammation and increase of bactericidal activity in the cecal ligation and puncture (CLP) experimental model [39]. Anti-allergic effects were detected in ovalbumin challenge in RBL-2H3 mast cells and ovalbumin sensitized the house mice (BALB/c) after cubebenoate treatment [22]. Furthermore, cubebenoate showed the anti-obesity effects in MDI-stimulated 3T3-L1 adipocytes through the inhibition of lipogenesis, stimulation of lipolysis, and suppression of inflammasome activation [30]. In this study, we investigated novel therapeutic effects of cubebenol in lipid metabolism. Our results suggest that cubebenol has anti-obesity effects similar to cubebenoate, although many additional mechanisms have been analyzed in this study.

Lipogenesis is a metabolic process in which the carbon precursors of Acetyl-CoA are converted into fatty acids and triglycerides [3]. Four key regulators tightly control this process: PPARγ, C/EBPs, aP2, and FAS. PPAR α and C/EBPs play a role as transcription factors that regulate the transcription of several genes, including aP2 and leptin, while aP2 and FAS are responsible for the transportation of fatty acids and the maturation of adipocytes [3]. Because of the above functions, these four factors are considered key targets in evaluating the anti-lipogenic effects of natural products or single compounds. Several single compounds derived from natural products, including 6,6-Bieckol, diallyl sulfide, and Platycodin D, have been proven effective in suppressing lipogenesis using these targets [8,9,10]. First, 6,6-Bieckol extracted from Eisenia bicyclis inhibited up to 60% fat accumulation at 50 μg/mL by downregulating the mRNA and protein expression of PPARγ, C/EBPα, aP2, and Fas in MDI-stimulated 3T3-L1 adipocytes [8]. Diallyl sulfide extracted from garlic inhibited fat accumulation by downregulating mRNA expression of PPARγ, C/EBPα, aP2, and Fas in the concentration of 5–50 mM for one–three hours in MDI-stimulated 3T3-L1 adipocytes [9]. In addition, platycodin D, extracted from *Platycodon grandiflorum*, inhibited up to 62.4% fat accumulation at 5 μM by downregulating the mRNA and protein expression of PPARγ, C/EBPα, aP2, and Fas in MDI-stimulated 3T3-L1 adipocytes [10]. This study examined the inhibitory effect of α-cubebenol on the lipogenesis of MDI-stimulated 3T3-L1 adipocytes using the analysis for PPAR γ, C/EBPs, aP2, and FAS expression. As results, α-cubebenol at 30 μg/mL effectively inhibited up to 75% fat accumulation and downregulated the mRNA expression of PPARγ, C/EBPα, aP2, and Fas in MDI-stimulated 3T3-L1 adipocytes. These results are similar to those of previous studies reporting the suppressive effects of 6,6-bieckol, diallyl sulfide, and platycodin D. Therefore, these results provide strong evidence that α-cubebenol have potential as a novel single compound with anti-lipogenic effects.

In addition, the inhibitory effects on lipolysis are considered another key therapeutic strategy to develop anti-obesity drugs, because triglycerides break down into glycerol and free fatty acids by the activation of lipases during this process. Perilipin, which is activated by cAMP, regulates the lipase activity [4]. Thus, the regulation of cAMP, activation of lipases and the release of glycerol have been widely investigated to identify natural products or single compounds with lipolysis-stimulating effects. Several compounds derived from natural products, including aculeatin and biflavone, were verified to have lipolysis-stimulating effects [11,12]. Aculeatin, extracted from *T. asiatica*, increased 2-deoxyglucose uptake and glycerol release in MDI-stimulated 3T3-L1 adipocytes [11], while biflavones increased the glycerol release in MDI-stimulated 3T3-L1 adipocytes [12]. In this study, the above factors investigated in previous studies were analyzed to evaluate the lipolysis stimulating effects of α-cubebenol in the MDI-stimulated 3T3-L1 adipocytes. The α-cubebenol treatment induced an increase in glycerol release, upregulation of cAMP concentration, adenylate cyclase expression, and activation of the cAMP downstream signaling pathway. In particular, the glycerol release induced by an α-cubebenol treatment was greater than those of the aculeatin and biflavone-treated cells, even though other factors are not directly comparable. Therefore, these results provide strong evidence that α-cubebenol has a high potential to be developed as a novel compound with lipolysis-stimulating effects.

Meanwhile, this study examined the inhibitory effects of α-cubebenol on the expression of iNOS, COX-2, inflammatory cytokines, and inflammasome expression, which are crucial for the maturation of IL-1β [3,5,6,7]. α-Cubebenol inhibited the expression of the iNOS and COX-2 proteins and the mRNA expression of the NF-κB, inflammatory cytokines, including IL-6, TNF-α, and IL-1β in the MDI-stimulated 3T3-L1 adipocytes. Moreover, α-cubebenol effectively inhibited the expression of NLRP3, ASC, and cleaved cas-1, which are the main components of inflammasome. The above results were similar to previous studies that investigated the anti-inflammatory effects of several single compounds. Allin, an extract from garlic, suppressed the phosphorylation of ERK1/2 and the expression of the IL-6, TNF- α, MCP-1 mRNA, and proteins in MDI-stimulated 3T3-L1 adipocyte [14]. In addition, saccharin suppressed the NO concentration, inflammatory cytokines, and NF-κB pathway by downregulating the expression of iNOS and COX-2 while increasing the protein expression of IκB [15]. In most studies, the suppression of adipose-derived inflammation is considered an important indicator for evaluating the anti-obesity effects of bioactive compounds, because it was accompanied by a key molecular mechanism during the inhibition of lipogenesis and the stimulation of lipolysis [5,6,7].

Finally, OT has been known as a saturated derivative of lipstatin firstly isolated from the *Streptomyces toxytricini* [38]. It specifically inhibits the gastric and pancreatic lipase, which hydrolyses the triglyceride into monoglyceride and free fatty acid [40]. Additionally, OT partially participates in the process of inhibiting dietary fat absorption [41]. Based on these mechanisms, OT induces the promotion of weight loss, reducing blood pressure, and preventing the onset of type 2 diabetes in human [42,43]. Furthermore, this compound stimulates anti-obesity effects including the inhibition of lipogenesis and stimulation of lipolysis in several adipocytes [44,45,46]. Because of these characterizations, OT is being applied to various studies in order to verify the anti-obesity effects of a compound in vivo and in vitro. In the present study, the OT treated group was used as a positive control group during the process of analyzing the anti-obesity effect of α-cubebenol. Among all analyzed factors, some such as PPARγ, Cas-1, TNFα, NF-κB, and CPT were measured at similar levels between the OT treated group and α-cubebenol treated group. However, most factors including lipogenesis regulators (C/EBPα, aP2 and FAS), lipolysis regulators (cAMP, AC, PDE4, ATGL, perilipin, HSL and free glycerol), inflammation factors (COX-2, NLRP3, ASC and IL-1β), and β-oxidation factors (ACADs, ACO and ATPCL) were higher in the α-cubebenol treated group than in the OT treated group, while a reversed pattern was detected on iNOS and IL-6 levels.

## 5. Conclusions

**Figure 9:**
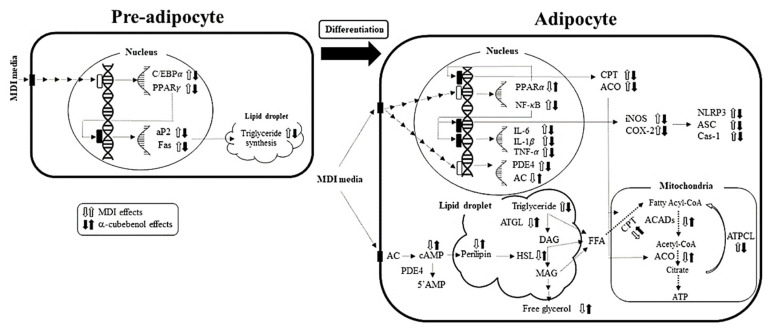
The proposed mechanism of α-cubebenol on anti-obesity effects in pre-adipocytes and adipocytes. During differentiation from pre-adipocytes to adipocytes, response stimulated by MDI are inhibited or promoted by α-cubebenol treatment.

This study examined the therapeutic effects and molecular mechanism of α-cubebenol on the lipogenesis, lipolysis, and adipose-derived inflammation in 3T3-L1 adipocytes. In pre-adipocytes, α-cubebenol suppressed MDI-stimulated lipogenesis by down-regulating the transcription of PPARγ and C/EBPα, which eventually resulted in suppression of triglyceride synthesis through the inhibition of both aP2 and Fas proteins. In mature adipocytes, α-cubebenol decreased the expression level of inflammatory cytokines and regulatory proteins as well as inflammasome protein through the regulation of NF-κB transcription factors, while it suppressed the expression of β-oxidation related proteins via the regulation of PPARα transcription factors. Additionally, the regulation of the lipolytic proteins expression and the release of free glycerol were induced by treatment of α-cubebenol in lipid droplet (Figure 9). The results provide scientific evidence that α-cubebenol inhibits lipogenesis and adipose-derived inflammation while it stimulates lipolysis and β-oxidation. The results further suggest that α-cubebenol suppresses inflammasome activation and the expression of inflammatory cytokines in 3T3-L1 adipocytes. On the other hand, additional studies of the molecular mechanisms in obese animal models will be needed to clarify the role of α-cubebenol as a lipogenesis inhibitor, lipolysis stimulator, and inflammasome activation inhibitor.

## Data Availability

The datasets generated during and analyzed during the current study are available from the corresponding author on reasonable request.

## Supplementary Materials

The following are available online at https://www.mdpi.com/article/10.3390/biom11111650/s1, Figure S1: Schematic diagram of the lipogenesis and lipolysis procedure, Figure S2: Determination of the optimal concentration of CN and cytotoxicity assessment, Figure S3: Apoptosis analysis of 3T3-L1 pre-adipocytes, Table S1: Primer sequences for RT-PCR, Table S2: Antibodies list for Western blot analyses.

## References

[1] Luo L., Liu M. (2016). Adipose tissue in control of metabolism. J. Endocrinol..

[2] Saponaro C., Gaggini M., Carli F., Gastaldelli A. (2015). The subtle balance between lipolysis and lipogenesis: A critical point in metabolic homeostasis. Nutrients.

[3] McArdle M., Finucane O., Connaughton R., McMorrow A., Roche H. (2013). Mechanisms of obesity-induced inflammation and insulin resistance: Insights into the emerging role of nutritional strategies. Front. Endocrinol..

[4] Lass A., Zimmermann R., Oberer M., Zechner R. (2011). Lipolysis—A highly regulated multi-enzyme complex mediates the catabolism of cellular fat stores. Prog. Lipid Res..

[5] Kershaw E.E., Flier J.S. (2004). Adipose tissue as an endocrine organ. J. Clin. Endocrinol. Metab..

[6] Coelho M., Oliveira T., Fernandes R. (2013). Biochemistry of adipose tissue: An endocrine organ. Arch. Med. Sci..

[7] Adamczak M., Wiecek A. (2013). The adipose tissue as an endocrine organ. Semin. Nephrol..

[8] Kwon T.H., Wu Y.X., Kim J.S., Woo J.H., Park K.T., Kwon O.J., Seo H.J., Kim T., Park N.H. (2015). 6,6′-Bieckol inhibits adipocyte differentiation through downregulation of adipogenesis and lipogenesis in 3T3-L1 cells. J. Sci. Food Agric..

[9] Wei T.C., Chi H.W., Chin L.H. (2015). *Diallyl trisulphide* inhibits adipogenesis in 3T3-L1 adipocytes through lipogenesis, fatty acid transport, and fatty acid oxidation pathways. J. Funct. Foods.

[10] Lee E.J., Kang M., Kim Y.S. (2012). Platycodin D inhibits lipogenesis through AMPKα-PPARγ2 in 3T3-L1 cells and modulates fat accumulation in obese mice. Planta Med..

[11] Watanabe A., Kato T., Ito Y., Yoshida I., Harada T., Mishima T., Fujita K., Watai M., Nakagawa K., Miyazawa T. (2014). Aculeatin, a coumarin derived from *Toddalia asiatica* (L.) Lam., enhances differentiation and lipolysis of 3T3-L1 adipocytes. Biochem. Biophys. Res. Commun..

[12] Dell’Agli M., Bosisio E. (2002). Biflavones of *Ginkgo biloba* stimulate lipolysis in 3T3-L1 adipocytes. Planta Med..

[13] Chang C.C., Lin K.Y., Peng K.Y., Day Y.J., Hung L.M. (2016). Resveratrol exerts anti-obesity effects in high-fat diet obese mice and displays differential dosage effects on cytotoxicity, differentiation, and lipolysis in 3T3-L1 cells. Endocr. J..

[14] Quintero-Fabián S., Ortuño-Sahagún D., Vázquez-Carrera M., López-Roa R.I. (2013). Alliin, a garlic (*Allium sativum*) compound, prevents LPS-induced inflammation in 3T3-L1 adipocytes. Mediat. Inflamm..

[15] Kim H.L., Ha A.W., Kim W.K. (2020). Effect of saccharin on inflammation in 3T3-L1 adipocytes and the related mechanism. Nutr. Res. Pract..

[16] Kim K.J., Lee B.Y. (2012). Fucoidan from the sporophyll of *Undaria pinnatifida* suppresses adipocyte differentiation by inhibition of inflammation-related cytokines in 3T3-L1 cells. Nutr. Res..

[17] Tsai C.W., Liu K.L., Lin Y.R., Kuo W.C. (2014). The mechanisms of carnosic acid attenuates tumor necrosis factor-α-mediated inflammation and insulin resistance in 3T3-L1 adipocytes. Mol. Nutr. Food Res..

[18] Lee M.S., Kim C.T., Kim I.H., Kim Y. (2011). Effects of capsaicin on lipid catabolism in 3T3-L1 adipocytes. Phytother. Res..

[19] Chin L.H., Gow C.Y. (2007). Effects of capsaicin on induction of apoptosis and inhibition of adipogenesis in 3T3-L1 cells. J. Agric. Food Chem..

[20] Isa Y., Miyakawa Y., Yanagisawa M., Goto T., Kang M.S., Kawada T., Morimitsu Y., Kubota K., Tsuda T. (2008). 6-Shogaol and 6-gingerol, the pungent of ginger, inhibit TNF-alpha mediated downregulation of adiponectin expression via different mechanisms in 3T3-L1 adipocytes. Biochem. Biophys. Res. Commun..

[21] Lee Y.J., Shim J.W., Lee Y.J., Park Y.H., Lee H.Y., Kim S.D., Choi Y.W., Bae Y.S. (2009). Identification of a novel compound that stimulates intracellular calcium increase and CXCL8 production in human neutrophils from *Schisandra chinensis*. Biochem. Biophys. Res. Commun..

[22] Lee K.P., Kang S., Park S.J., Kim J.M., Lee J.M., Lee A.Y., Chung H.Y., Choi Y.W., Lee Y.G., Im D.S. (2015). Anti-allergic effect of α-cubebenoate isolated from *Schisandra chinensis* using in vivo and in vitro experiments. J. Ethnopharmacol..

[23] Lee Y.J., Park S.Y., Kim S.G., Park D.J., Kang J.S., Lee S.J., Yoon S., Kim Y.H., Bae Y.S., Choi Y.W. (2010). Identification of a novel compound that inhibits iNOS and COX-2 expression in LPS-stimulated macrophages from *Schisandra chinensis*. Biochem. Biophys. Res. Commun..

[24] Park S.Y., Park T.G., Lee S.J., Bae Y.S., Ko M.J., Choi Y.W. (2014). α-Iso-cubebenol inhibits inflammation-mediated neurotoxicity and amyloid beta 1-42 fibril-induced microglial activation. J. Pharm. Pharmacol..

[25] Park S.Y., Park D.J., Kim Y.H., Kim Y., Choi Y.W., Lee S.J. (2011). *Schisandra chinensis* α-iso-cubebenol induces heme oxygenase-1 expression through PI3K/Akt and Nrf2 signaling and has anti-inflammatory activity in *Porphyromonas gingivalis* lipopolysaccharide-stimulated macrophages. Int. Immunopharmacol..

[26] Park S.Y., Kim D.Y., Kang J.K., Park G., Choi Y.W. (2014). Involvement of activation of the Nrf2/ARE pathway in protection against 6-OHDA-induced SH-SY5Y cell death by α-iso-cubebenol. Neurotoxicology.

[27] Song S.H., Choi S.M., Kim J.E., Sung J.E., Lee H.A., Choi Y.H., Bae C.J., Choi Y.W., Hwang D.Y. (2017). α-Iso-cubebenol alleviates scopolamine-induced cognitive impairment by repressing acetylcholinesterase activity. Neurosci. Lett..

[28] Kim J.E., Kim S.G., Goo J.S., Park D.J., Lee Y.J., Hwang I.S., Lee H.R., Choi S.I., Lee Y.J., Oh C.H. (2012). The α-iso-cubebenol compound isolated from *Schisandra chinensis* induces p53-independent pathway-mediated apoptosis in hepatocellular carcinoma cells. Oncol. Rep..

[29] Lee S.K., Kim S.D., Kook M., Lee H.Y., Park J.S., Park Y.H., Kang J.S., Jung W.J., Choi Y.W., Bae Y.S. (2012). Therapeutic effects of α-iso-cubebenol, a natural compound isolated from the *Schisandra chinensis* fruit, against sepsis. Biochem. Biophys. Res. Commun..

[30] Bae S.J., Kim J.E., Choi Y.J., Lee S.J., Gong J.E., Choi Y.W., Hwang D.Y. (2020). Novel function of alpha-Cubebenoate derived from *Schisandra chinensis* as lipogenesis inhibitor, lipolysis stimulator and inflammasome suppressor. Molecules.

[31] Park H.J., Cho J.Y., Kim M.K., Koh P.O., Cho K.W., Kim C.H., Lee K.S., Chung B.Y., Kim G.S., Cho J.H. (2012). Anti-obesity effect of *Schisandra chinensis* in 3T3-L1 cells and high fat diet-induced obese rats. Food Chem..

[32] Jeong Y.S., Jung H.K., Cho K.H., Youn K.S., Hong J.H. (2011). Anti-obesity effect of grape skin extract in 3T3-L1 adipocytes. Food Sci. Biotechnol..

[33] Livak K.J., Schmittgen T.D. (2001). Analysis of relative gene expression data using real-time quantitative PCR and the 2^−ΔΔCT^ method. Methods.

[34] Ekiert R.J., Szopa A., Ekiert H., Krzek J., Dzik E. (2013). Analysis of lignans in *Schisandra chinensis* fruits, leaves, biomasses from in vitro cultures and food supplements. J. Funct. Foods.

[35] Mocan A., Crisan G., Vlase L., Crissan O., Vodnar D.C., Raita O., Gheldiu A.M., Toiu A., Oprean R., Tilea I. (2014). Comparative studies on polyphenolic composition, antioxidant and antimicrobial activities of *Schisandra chinensis* leaves and fruits. Molecules.

[36] Szopa A., Ekiert H. (2016). The importance of applied light quality on the production of lignans and phenolic acids in *Schisandra chinensis* (Turcz.) Baill. cultures in vitro. Plant Cell Tissue Organ Cult..

[37] Kook M.S., Lee S.K., Kim S.D., Lee H.Y., Hwang J.S., Choi Y.W., Bae Y.S. (2015). Anti-septic activity of α-cubebenoate isolated from *Schisandra chinensis*. BMB Rep..

[38] Barbier P., Schneider F. (1987). Syntheses of tetrahydrolipstatin and absolute configuration of tetrahydrolipstatin and lipstatin. Helv. Chim..

[39] Kang S.R., Lee K.P., Park S.J., Noh D.Y., Kim J.M., Moon H.R., Lee Y.G., Choi Y.W., Im D.S. (2014). Identification of a novel anti-inflammatory compound, α-cubebenoate from *Schisandra chinensis*. J. Ethnopharmacol..

[40] Guerciolini R. (1997). Mode of action of orlistat. Int. J. Obes. Relat. Metab. Disord..

[41] Ballinger A., Peikin S.R. (2002). Orlistat: Its current status as an anti-obesity drug. Eur. J. Pharmacol..

[42] Padwal R., Li S.K., Lau D.C., Padwal R.S. (2004). Long-term pharmacotherapy for obesity and overweight. Cochrane Database Syst. Rev..

[43] Gillies C.L., Abrams K.R., Lambert P.C., Cooper N.J., Sutton A.J., Hsu R.T., Khunti K. (2007). Pharmacological and lifestyle interventions to prevent or delay type 2 diabetes in people with impaired glucose tolerance: Systematic review and meta-analysis. BMJ (Clin. Res. Ed.).

[44] Hussain S., Rehman A.U., Luckett D.J., Blanchard C.L., Obied H.K., Strappe P. (2020). Phenolic compounds with antioxidant properties from canola meal extracts inhibit adipogenesis. Int. J. Mol. Sci..

[45] Li C., Dong X., Du W., Shi X., Chen K., Zhang W., Gao M. (2020). LKB1-AMPK axis negatively regulates ferroptosis by inhibiting fatty acid synthesis. Sig. Transduct. Target Ther..

[46] Ding Y., Gu Z., Wang Y., Wang S., Chen H., Zhang H., Chen W., Chen Y.Q. (2017). Clove extract functions as a natural fatty acid synthesis inhibitor and prevents obesity in a mouse model. Food Funct..

