# Supplementary material for: Anti-Obesity Effect of α-Cubebenol Isolated from Schisandra chinensis in 3T3-L1 Adipocytes
Source: Biomolecules. 2021 Nov 8;11(11):1650. doi: 10.3390/biom11111650 (PMC8615670; doi:10.3390/biom11111650)
Supplement: Supplementary file 1 [file biomolecules-11-01650-s001.zip › biomolecules-1391696-supplementary.pdf]

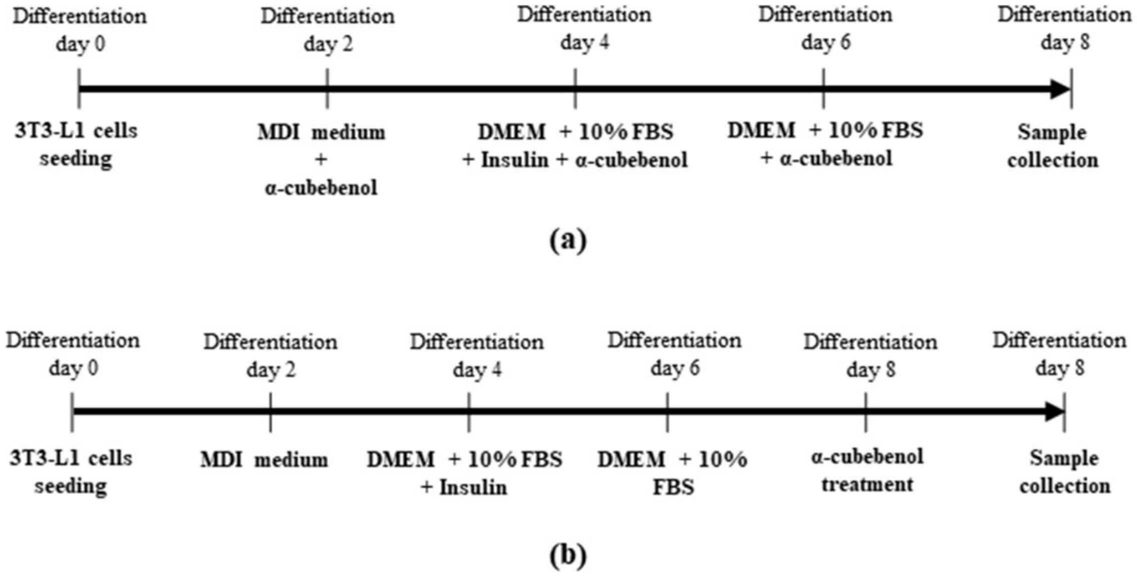


**Figure S1.** Schematic diagram of the lipogenesis and lipolysis procedure. (**a**) Schedule for the inhibitory effects of $\alpha$-cubebenol on lipogenesis in 3T3-L1 cells. (**b**) Schedule for the stimulatory effect of $\alpha$-cubebenol on lipolysis in 3T3-L1 cells.

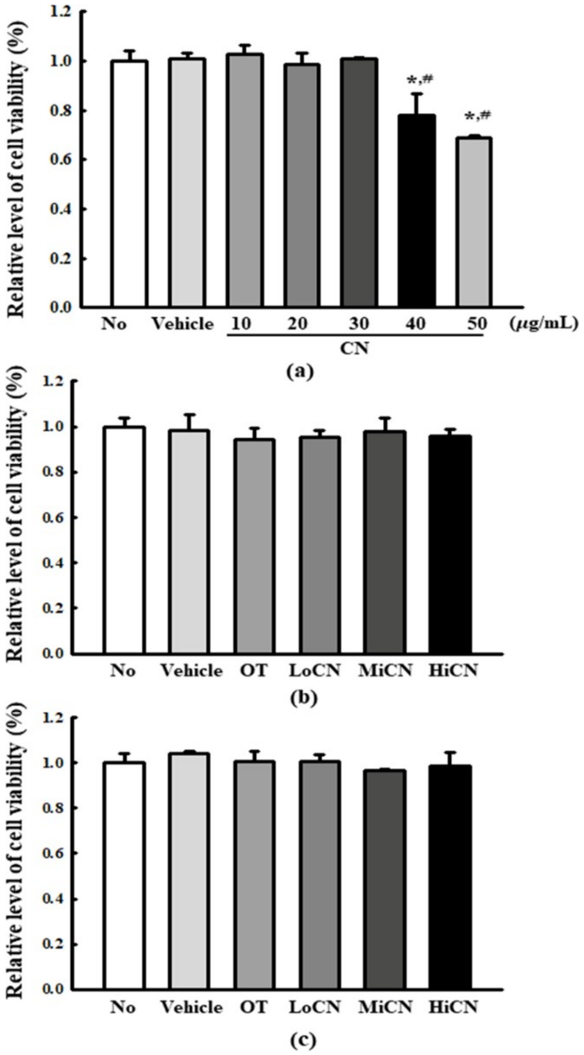


**Figure S2.** Determination of the optimal concentration of CN and cytotoxicity assessment. (**a**) Viability of 3T3-L1 pre-adipocytes against *α*-cubebenol against six different doses of *α*-cubebenol (10, 20, 30, 40, and 50 μg/mL) for 24 h. (**b**) and (**c**) Viability of 3T3-L1 pre-adipocytes against *α*-cubebenol. After the treatment of 7.5, 15, and 30 μg/mL of *α*-cubebenol for 48 h (**b**) and 72 h (**c**). The cell viability was determined using the MTT assay. Three wells per group were used in the MTT assay, and the optical density was measured in triplicates. The data are reported as the means ± SD.

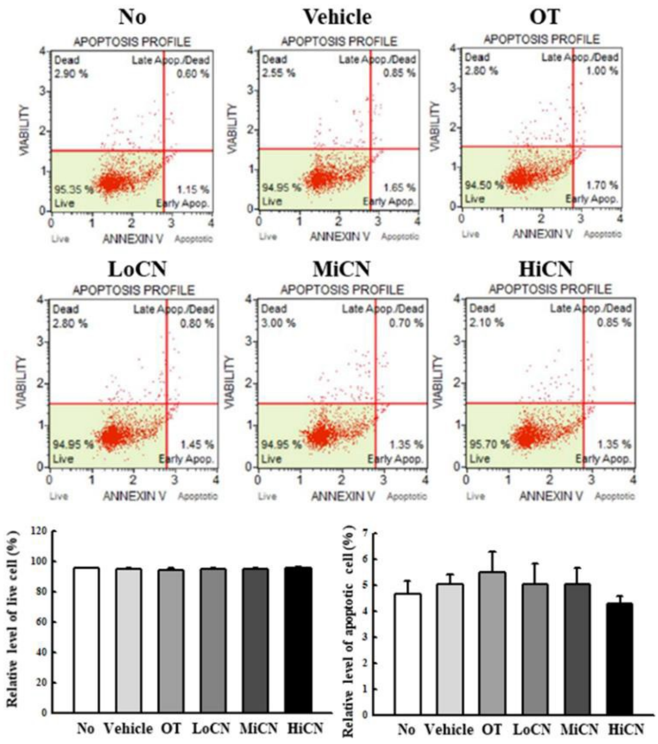
No
Vehicle
OT
LoCN
MiCN
HiCN
APOPTOSIS PROFILE
VIABILITY
ANNEXIN V
Dead
Late Apop./Dead
Live
Early Apop.
Apoptotic
2.90 %
0.60 %
95.35 %
1.15 %
2.55 %
0.85 %
94.95 %
1.65 %
2.80 %
1.00 %
94.50 %
1.70 %
2.80 %
0.80 %
94.95 %
1.45 %
3.00 %
0.70 %
94.95 %
1.35 %
2.10 %
0.85 %
95.70 %
1.35 %
Relative level of live cell (%)
Relative level of apoptotic cell (%)

(a)

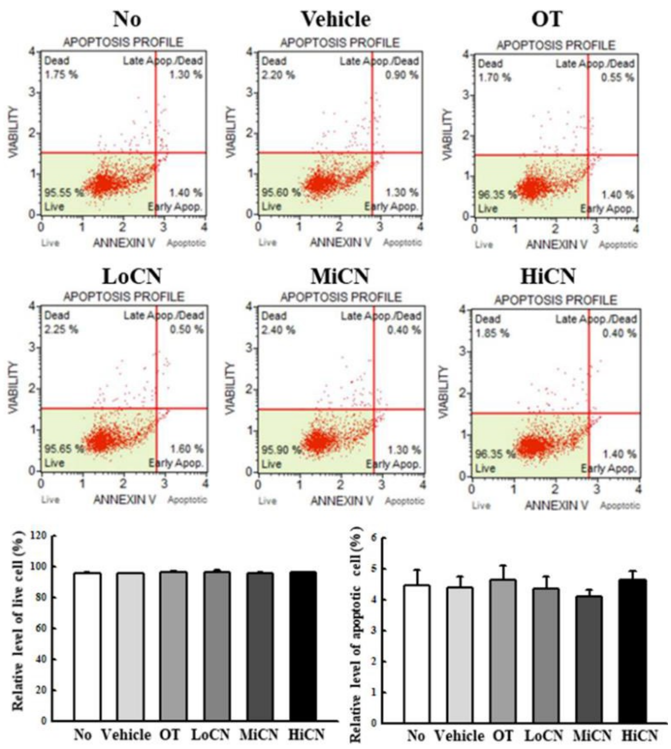
No
Vehicle
OT
LoCN
MiCN
HiCN
APOPTOSIS PROFILE
VIABILITY
ANNEXIN V
Dead
Late Apop./Dead
Live
Early Apop.
Apoptotic
1.75 %
1.30 %
95.55 %
1.40 %
2.20 %
0.90 %
95.60 %
1.30 %
1.70 %
0.55 %
96.35 %
1.40 %
2.25 %
0.50 %
95.65 %
1.60 %
2.40 %
0.40 %
95.90 %
1.30 %
1.85 %
0.40 %
96.35 %
1.40 %
Relative level of live cell (%)
Relative level of apoptotic cell (%)

(b)

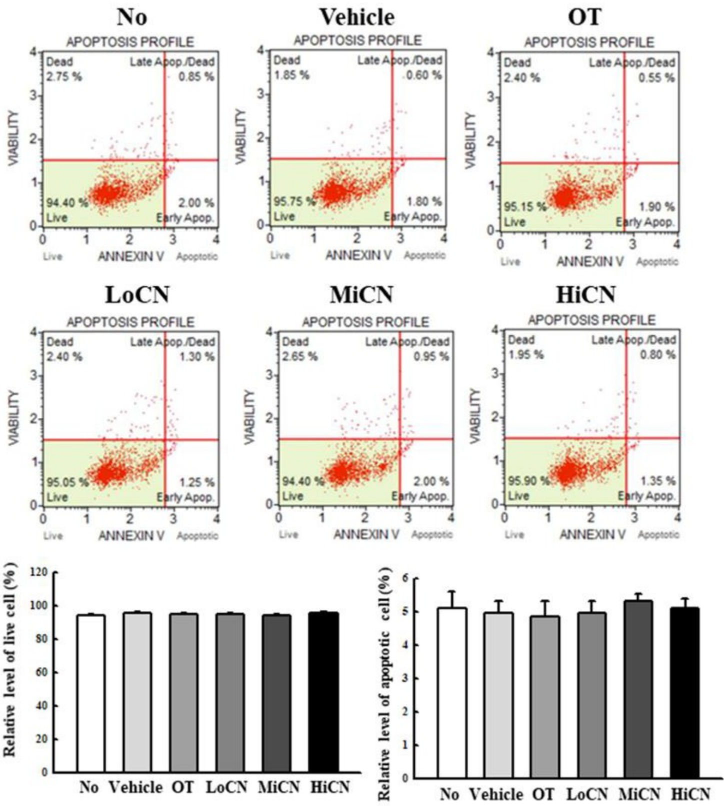


(c)

**Figure S3.** Apoptosis analysis of 3T3-L1 pre-adipocytes. After treatment with $\alpha$-cubebenol or OT for 24 h (**a**), 48 h (**b**), 72 h (**c**), the distribution of apoptotic cells was analyzed subsequent to staining with annexin V and 7-AAD. Initial cell population gating was placed on cell size vs. Annexin V. Subsequently, the most obvious debris was gated out from the total cell population. Two to three wells per group were used for Annexin V staining, and the number of dead cells and live cells was measured in duplicate. Data are reported as the mean ± SD. Abbreviations: 7-AAD, 7-aminoactinomycin D.

**Table S1.** Primer sequences for RT-PCR.

| Primer name | Sequence (from 5' to 3') | Product size (bp) |
|---|---|---|
| PPARγ ForwardReverse | CCC CTG CTC CAG GAG ATC TAC<br>GCA ATC AAT AGA AGG AAC ACG TTG T | 80 |
| C/EBPα Forward Reverse | GAC CAT TAG CCT TGT GTG TAC TGT ATGTGG ATC GAT TGT GCT TCA AGT T | 71 |
| Fas ForwardReverse | CAA GCA GAA TTT GTC CAC CTT TAATCT CTA GAG GGC TTG CAC CAA | 128 |
| aP2 ForwardReverse | TGG ACT TCA GAG GCT CAT AGC ACCT TCG AGG AGG AGC TGT CTT | 72 |
| Adenylyl cyclaseForward Reverse | CGG AGG TTG CTG CAT AAC ATT<br>ACA CAT TCA CAC GAC TGG TAG TAC AG | 106 |
| PDE4 Forward Reverse | TTC CCT CAT CAC CCT ACC TAT CAGGC AGA GCA GAG GTT CAA GCT | 98 |
| CPT1 Forward Reverse | GGC AGA GCA GAG GTT CAA GCTGCC AGC GCC CGT CAT | 81 |
| PPARα Forward Reverse | TGG CAA AAG GCA AGG AGA AG<br>CCC TCT ACA TAG AAC TGC AAG GTT T | 104 |
| TNF-α Forward Reverse | CCT GTA GCC CAC GTC GTA GCTTG ACC TCA GCG CTG ACT TG | 374 |
| IL-6 Forward Reverse | TTG GGA CTG ATG TTG TTG ACA<br>TCA TCG CTG TTG ATA CAA TCA GA | 200 |
| IL-1*β* Forward Reverse | CAG TTC TGC CAT TGA CCA T<br>TCT CAC TGA AAC TCA GCC GT | 218 |
| NF-*κ*B Forward Reverse | GTA ACA GCA GGA CCC AAG GAAGC CCC TAA TAC ACG CCT CT | 501 |
| β-actin Forward Reverse | TGG AAT CCT GTG GCA TCC ATG AAA CTAA AAC GCA GCT CAG TAA CAG TCC G | 349 |

**Table S2.** Antibodies list for Western blot analyses.

| Name | Cat. No. | Company |
|---|---|---|
| Anti-iNOS | PA3-030A | Thermo Fisher Scientific, MA, USA |
| Anti-Cox-2 | 4842 | Cell Signaling, Danvers, MA, USA |
| Anti-ASC | 67824 | Cell Signaling, Danvers, MA, USA |
| Anti-Cas-1 | 24232 | Cell Signaling, Danvers, MA, USA |
| Anti-NLRP3 | 15101 | Cell Signaling, Danvers, MA, USA |
| Anti-ATGL | 2138s | Cell Signaling, Danvers, MA, USA |
| Anti-Perilipin | 9349s | Cell Signaling, Danvers, MA, USA |
| Anti-p-Perilipin | 9621s | Cell Signaling, Danvers, MA, USA |
| Anti-HSL | 4107s | Cell Signaling, Danvers, MA, USA |
| Anti-p-HSL | PA5-64494 | Thermo Fisher Scientific, MA, USA |
| Anti-ACADs | PA5-54580 | Thermo Fisher Scientific, MA, USA |
| Anti-ACO1 | PA5-41753 | Thermo Fisher Scientific, MA, USA |
| Anti-ATP Citrate Lyase | #4332s | Cell Signaling, Danvers, MA, USA |
| Anti-p-ATP Citrate Lyase | #4331s | Cell Signaling, Danvers, MA, USA |
| Anti-*β*actin | 87809 | Cell Signaling, Danvers, MA, USA |
